# Arterial tortuosity syndrome in a newborn

**DOI:** 10.1007/s00247-025-06256-9

**Published:** 2025-05-07

**Authors:** Pietro G. Lacaita, Gudrun M. Feuchtner

**Affiliations:** Department of Radiology, Medical University Innsbruck, Anichstrasse 35, Innsbruck, A-6020 Austria

**Keywords:** Computed tomography, Computed tomography angiography, Congential heart disease, Imaging


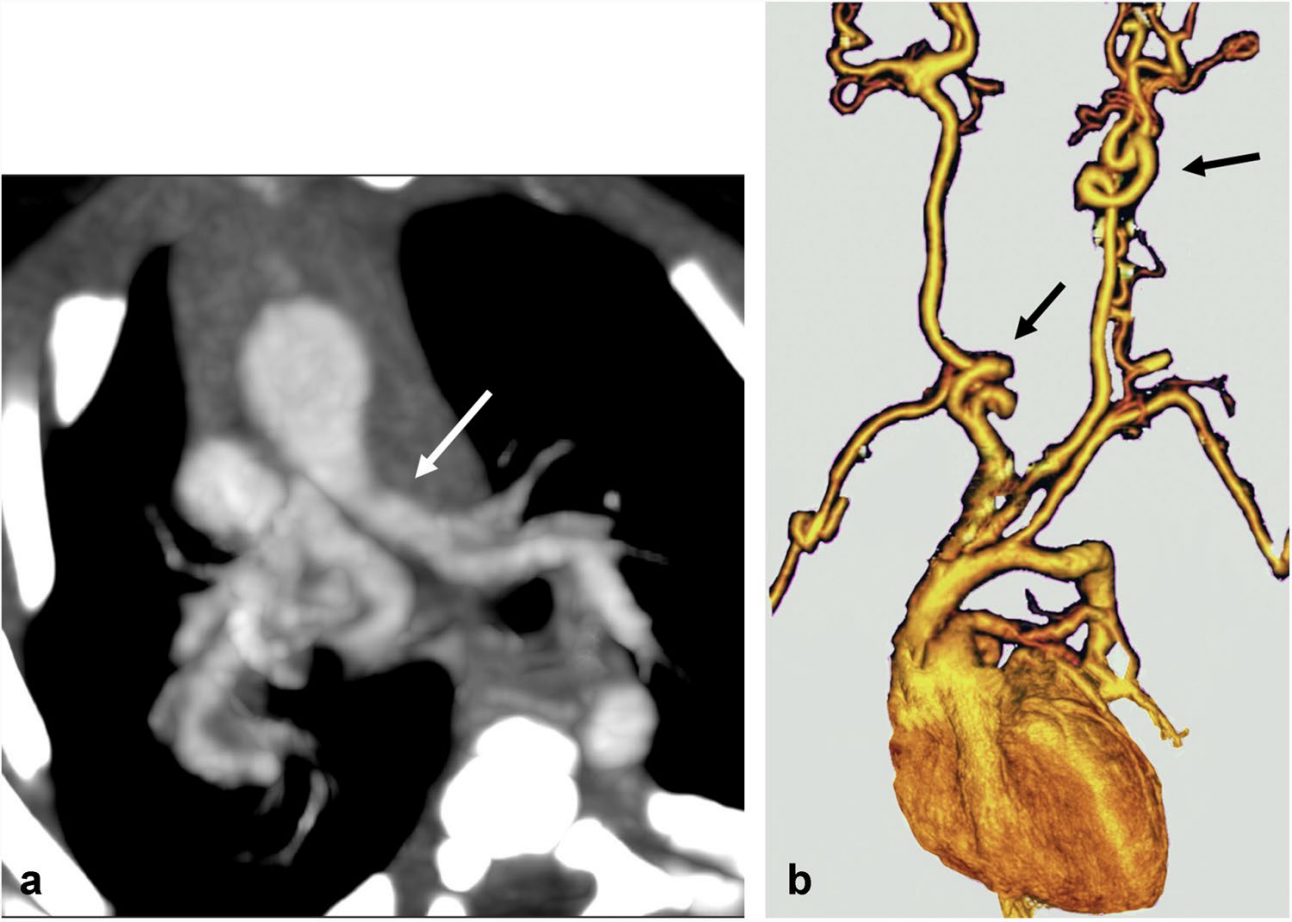


A 2-day-old girl presented with cutis laxa, frequent vomiting, and infant respiratory distress syndrome, necessitating neonatal intensive care. Echocardiography showed aortic elongation. An anteroposterior chest radiograph demonstrated an enlarged cardiomediastinal silhouette and a retrocardiac lucency due to a large diaphragmatic hernia, which was confirmed by computed tomography angiography (CTA).

Axial CTA (**a)** showed an inverted V-shaped configuration of the pulmonary artery bifurcation (*arrow*), along with tortuosity and narrowing of the left main pulmonary artery (*arrow*). Three-dimensional reconstructions of the vertebral arteries (**b**) showed significant arterial twisting (*arrows* in** b**).

Genetic profiling confirmed arterial tortuosity syndrome, a rare connective tissue disorder caused by a mutation in the *SLC2A10* gene. Radiological features were vascular twisting and pulmonary stenosis. Additional associated abnormalities with the syndrome include kyphoscoliosis, chest wall deformities, hernias (inguinal and/or diaphragmatic), vascular aneurysms, and skin laxity.

## Data Availability

No datasets were generated or analysed during the current study.

